# Urethral Diverticulum following Polypropylene Mesh Midurethral Slings: A Literature Review

**DOI:** 10.1155/2020/6761581

**Published:** 2020-05-11

**Authors:** Elizabeth Pacer, Omar Felipe Duenas-Garcia, Lekha Hota, Kristan Hornsby, Robert Shapiro

**Affiliations:** Department of Obstetrics & Gynecology, West Virginia University School of Medicine, Morgantown, WV 26506, USA

## Abstract

**Aims:**

It is currently unknown whether an association exists between polypropylene mesh and urethral diverticulum formation following placement of polypropylene midurethral slings (MUS) for the treatment of stress urinary incontinence (SUI). We aimed to examine the literature associating MUS with the occurrence of urethral diverticula.

**Methods:**

Multiple online research databases, including PubMed, Google Scholar, EBSCOhost, and the Cochrane Library, were searched, from January 2019 to February 2019, for evidence related to the occurrence of urethral diverticula following polypropylene MUS procedures.

**Results:**

Four case reports were published demonstrating the occurrence of urethral diverticula following the use of polypropylene mesh for surgical treatment of SUI. Subjects of these cases were menopausal and had an elevated body mass index (BMI), recurrent urinary tract infections (UTIs), autoimmune conditions, or prior pelvic floor surgeries. A thorough urologic workup, including imaging prior to sling placement, was not always performed.

**Conclusion:**

No clear association exists between polypropylene MUS placement and subsequent urethral diverticulum formation. Factors that diminish polypropylene mesh biocompatibility include elevated BMI, menopause, recurrent UTIs, prior pelvic surgeries, and preexisting medical conditions. Symptoms associated with urethral diverticula should prompt a complete urologic workup prior to MUS placement.

## 1. Introduction

A urethral diverticulum is an outpouching of urethral mucosa into surrounding tissues [1]. The outpouching can form anywhere along the urethra and is most common at the mid or distal third of the urethra [1]. The prevalence of urethral diverticula is estimated around 1-5% of the general population with an incidence less than 0.02 percent per year [1]. Urethral diverticula are commonly diagnosed between the third and fifth decades of life [2]. The imaging modality of choice for diagnosis is magnetic resonance imaging, as it accurately enables both the detection of the diverticulum and the information necessary for proper surgical treatment [3]. Other diagnostic methods include voiding cystourethrography, double-balloon urethrography, and ultrasound [3].

While the etiology of urethral diverticula continues to be debated, trauma, inflammation, obstruction, and congenital malformations are the most likely causes [4–7]. Obstruction of the periurethral glands with subsequent dilation and rupture into the lumen of the urethra is thought to be the leading cause of urethral diverticulum formation, while congenital malformation is the least common etiology and seen only in the pediatric population [8]. Once formed, the diverticula then epithelializes [9]. Inflammation usually occurs due to repeated infections at the site of diverticulum formation [4]. Trauma as a potential etiology may occur from childbirth or from iatrogenic or urethral instrumentation [4].

Synthetic MUS are the most common primary surgical treatment for SUI and have been designated as the standard of care by the American Urologic Association (AUA) and the American Urogynecologic Society (AUGS) [10]. In recent years, synthetic mesh has come under increased scrutiny by the Federal Drug Administration (FDA) due to possible concerns over patient safety [11].

Synthetic mesh, as with any foreign material, initiates an inflammatory response within the body [12–17]. Polypropylene mesh is a large pore, monofilament mesh used primarily in synthetic MUS and pelvic organ prolapse surgeries such as sacrocolpopexy [12–15]. This particular type of mesh has the advantage of causing less tissue inflammation compared to other meshes with smaller pores [12–17]. In one study examining foreign body response, polypropylene mesh caused less tissue fibrosis and less inflammation when compared to the other types of implants [13]. The large pore size and low inflammatory response allow for better soft tissue ingrowth into the mesh [18]. However, this soft tissue ingrowth may potentially cause an obstruction of the periurethral glands and lead to the formation of urethral diverticula.

Due to the frequent use of polypropylene mesh for the treatment of SUI, we conducted a literature review to examine the occurrence of urethral diverticula in patients who underwent MUS placement for SUI. When such occurrences were found, the cases were compared to extricate commonalties among those affected patients.

## 2. Literature Search

Online databases including PubMed, Google Scholar, EBSCOhost, and the Cochrane Library were utilized to identify cases in the literature. Search terms included polypropylene, mesh, stress incontinence repair, urethral sling, inflammation, urethral diverticulum, and tension free vaginal tape. As of January 2019, four case reports exist that identify diverticula following mesh-based sling procedure. This search also examined previous literature reviews, case reports, and retrospective cohort studies regarding urethral diverticula in females, dating from the mid-twentieth century onward.

## 3. Review of Cases

To date, four known cases exist in the literature in which urethral diverticula occurred in patients who underwent mesh-based sling procedures for SUI [3, 19–21]. The first case published in July 2006 involves a 43-year-old G5P5 with gravidity (G) meaning number of pregnancies and parity (P) meaning number of deliveries. She had a past surgical history significant for two cesarean sections and an anterior colporrhaphy. She underwent a retropubic MUS for SUI. Postoperatively, her incontinence improved, but persistent urinary dribbling developed a few months following the MUS placement. On presentation, significant findings included an overweight patient (BMI > 30 kg/m^2^) with a well-healed vaginal scar without evidence of cough leakage. Urinalysis showed no signs of infection. A slight obstructive urination pattern was seen on uroflow, and cystoscopy revealed a normal urethra without sign of mesh erosion. Additionally, bladder examination was normal. The patient's urinary dribbling persisted which led to a high suspicion for a urethral diverticulum. This facilitated a urinary workup which included a voiding cystourethrogram (VCUG). The VCUG revealed a communication of the urethra with a proximal outpouching of urethral mucosa consistent with a urethral diverticulum.

The patient underwent urethral diverticulectomy with concurrent urethrolysis, and the patient's urinary dribbling resolved postoperatively. Improvement of urinary flow also occurred, and no further complications were noted.

The next reported case published in 2007 consisted of a 53-year-old female with a three-year history of SUI who underwent a retropubic MUS procedure six months prior to seeking medical attention for postmicturition incontinence. A fluro-urodynamic study (FUDS) revealed an open bladder neck and proximal formation of a urethral diverticulum. She then underwent a takedown of the MUS, resection of the diverticulum, and a placement of an autologous pubovaginal sling at the level of the bladder neck. Postoperatively, her symptoms of postmicturition incontinence resolved.

The third case published in 2008 presented a 54-year-old patient with urinary frequency, urgency, and urge incontinence. She was using 1-2 incontinence pads per day. She described constant, vague vaginal pain and sharp urethral pain. Past medical/surgical history was significant for recurrent UTIs and a retropubic MUS for urodynamically confirmed SUI three years prior to presentation. Her operative report disclosed no urethral abnormalities or surgical complications peri- or postoperatively. Physical examination showed no signs of sling erosion or infection. No urethral masses or urethral hypermobility was appreciated. She then underwent FUDS indicating evidence of bladder outlet obstruction. Cystourethroscopy showed a wide ostium urethral diverticulum distal to the sling site. This patient subsequently underwent sling urethrolysis, transvaginal diverticulectomy, and repair of the urethral defect. She was observed for a year postoperatively and demonstrated improvement of symptoms.

The fourth case involved a 48-year-old G2P2 who presented in March 2016, with vaginal discharge, dysuria, and dyspareunia. Her BMI on presentation was >30 kg/m^2^. Her past medical history consisted of multiple sclerosis and Crohn's disease. Her surgical history was significant for two prior cesarean sections, bilateral tubal ligation, retropubic MUS, and cholecystectomy. Physical examination showed a small anterior vaginal wall outpouching near the urethra. Magnetic resonance imaging (MRI) revealed a cystic mass posterolateral to the urethra. In June 2016, she underwent urethrolysis of her previous MUS. No diverticulum was able to be identified at that time. The patient experienced persistent vaginal discharge, urinary dribbling, and history of dysuria. In 2018, the patient underwent transvaginal ultrasound which identified a urethral diverticulum. The diverticulum was excised, and the urethral defect was repaired using cadaveric pericardium. At three and six months postoperatively, the patient was completely asymptomatic. She denied any further urinary incontinence, dysuria, or discharge.

## 4. Discussion

Urethral diverticulum presents with nonspecific clinical presentations. Dysuria, dyspareunia, urinary incontinence, and recurrent urinary tract infections are a few of the symptoms that often facilitate further urinary workup leading to diagnosis [5, 22]. Clinical examination can reveal a cystic mass along the anterior vaginal wall that when palpated exudes pus from the urethra. Exam findings are often nonspecific which is why imaging is needed to confirm the diagnosis [5]. MRI is the preferential diagnostic test of urethral diverticula [3, 5, 22]. It has the highest tissue sensitivity and can even detect diverticula prior to the onset of symptoms [3, 22]. In three out of the four cases in which urethral diverticula occurred in women who underwent MUS placement, no MRI was performed prior to this procedure. As it cannot be concluded the diverticula formed after sling placement, other etiologies of diverticulum formation should be explored.

A biomaterial is one that contacts human tissue without eliciting harm. The extent to which a biomaterial can remain in the host organism for an extended period of time and perform the same function as the original structure is referred to as *biocompatibility* [23, 24]. In reference to implantable medical devices such as a MUS, the biocompatibility is affected by the qualities inherent to the host receiving the implant. Such qualities include age, sex, comorbid conditions, pharmacological status, general health, and physical limitations [23]. In a study examining the biocompatibility of polypropylene mesh, the vaginal smooth muscle proliferation was greater in patients of younger age, lower BMI, and absence of diabetes and smoking [12]. An increased rate of proliferation enables the mesh to be incorporated as part of the host environment faster and more effectively. In the case of inadequate biocompatibility, fibrotic tissue surrounds the mesh causing weakness and ultimately mesh failure [23].

Knowing that weakened tissue predisposes to diverticulum formation [4], the subjects in each of these cases had risk factors for weakened tissue and poor mesh biocompatibility. Elevated BMI puts more strain on the urethra and further weakens the pelvic floor. Higher intra-abdominal pressures are observed in patients with BMI greater than 30 kg/m^2^, and this stresses the urethra and surrounding tissues [24].

In the four cases in which a urethral diverticulum occurred in patients who had MUS, parity was provided for two of the four women. Case 1 presents a G5P5, for which two of her deliveries were cesarean sections, and case 4 presents a G2P2, who delivered via cesarean section for each birth. No information was provided regarding the vaginal deliveries, specifically if any assistance was required or trauma occurred. Multiparity is a factor that can contribute to pelvic floor weakness [25].

Urinary tract infections are common in females with urethral diverticula, as one study showed half its subjects with culture proven UTIs and another approximated that urinary tract infections are present in a third of women with diverticula [7, 26]. The urethral outpouching provides a nidus for bacterial colonization, often evident by recurrent infection with the same microbe [5]. This can lead to a blockage of the periurethral glands, a prominent factor in the pathogenesis of urethral diverticula [22, 27]. Recurrent UTIs are present in approximately 25% of patients with urethral diverticulum [27]. The subject of case 3 experienced recurrent UTIs, as did the patient described in case 4.

Menopause is another factor shown to influence the biocompatibility of implanted polypropylene mesh [12]. Estrogen receptors are dispersed throughout the lower urinary tract [28]. This makes the urethra especially sensitive to lower estrogen from menopause and could lead to tissue atrophy [28]. Cases 1 and 4 presented women in their fifth decade of life, and cases 2 and 3 presented women in their sixth decade of life. Further research is required to ascertain the age group at highest risk for diverticulum formation as these cases suggested women over age 40 may be at risk.

As inflammation serves a role in the pathogenesis of urethral diverticulum [9], patients who suffer from autoimmune conditions may be at a higher risk for diverticulum formation due to the presence of an already activated immune response. Case 4 consists of a woman who had Crohn's disease and multiple sclerosis, which are known to increase the body's state of inflammation.

Incontinence is yet another common finding in females who have urethral diverticulum [4]. The only medical history provided on case 2 is a 3-year history of urinary incontinence prior to presentation. The subject in case 3 also presented with a 3-year history of incontinence prior to diagnosis of her urethral diverticulum. As no imaging was performed or included prior to either patient's sling placement, it is plausible that the diverticulum was present prior to the mesh placement.

Incorporation of mesh into tissues is a complicated biochemical healing process. New blood vessels and collagen form around the mesh, with an increase in the type I to type III collagen ratio over time [29]. Adequate tissue ingrowth into the mesh results in superior biocompatibility and likely improved clinical performance. In patients with surgically placed polypropylene MUS, this is the likely outcome. However, poor biocompatibility causes a fibrotic capsule around the mesh [30]. The magnitude of this fibrotic response can result in chronic inflammation, mesh contracture, and pain due to pressure on ingrown nerves [31].

## 5. Conclusion

Although the etiology of urethral diverticula is debatable, it most commonly occurs due to obstruction of the periurethral glands and subsequent rupture into the lumen of the urethra. While it may be a theoretical complication that can occur with polypropylene MUS, this is unlikely given the limited number of reportable cases in the literature. Factors that diminish polypropylene mesh biocompatibility include elevated BMI, menopause, recurrent UTIs, prior pelvic surgeries, and preexisting medical conditions [12, 23, 24]. Symptoms associated with urethral diverticula should prompt a complete urologic workup prior to MUS placement.

## Additional Points

*Brief Summary*. The low number of urethral diverticulum cases after MUS placement suggests there is no association between polypropylene mesh and urethral diverticulum formation.
